# A mixed methods analysis of factors affecting antenatal care content: A Syrian case study

**DOI:** 10.1371/journal.pone.0214375

**Published:** 2019-03-25

**Authors:** Rima Mourtada, Christian Bottomley, Fiona Houben, Hyam Bashour, Oona M. R. Campbell

**Affiliations:** 1 Department of Epidemiology and Population Health, Faculty of Health Sciences, American University of Beirut, Beirut, Lebanon; 2 Department of Epidemiology and Population Health, London School of Hygiene and Tropical Medicine, London, United Kingdom; 3 Faculty of Health and Wellbeing, Canterbury Christ Church University, Canterbury, Kent, United Kingdom; 4 University of Damascus, Damascus, Syria; Makerere University, UGANDA

## Abstract

**Background:**

Maternity care services provide critical interventions aimed at improving maternal and newborn health. In this study, we examined determinants of antenatal care (ANC) content in Syria, together with changes over time.

**Methods:**

We analysed two national surveys conducted by the Central Bureau of Statistics in Damascus (PAPFAM 2001 and MICS 2006). Findings of this initial analysis led to a qualitative study on adequacy of antenatal care content in two Syrian governorates, Aleppo and Latakia in 2010, which in turn informed further quantitative analysis. The perspectives and practices of doctors, women, midwives and health officials were explored using in-depth interviews. A framework approach was used to analyse the data.

**Results:**

The quantitative analysis demonstrated that women’s education level, the type of health facility they attended and whether they had experienced health complications were important determinants of adequacy of ANC content received. The qualitative study revealed that additional factors related to supply side and demand side factors (e.g. organization of health services, doctors' selective prescription of ANC tests and women's selective uptake of those tests), influenced the quality of ANC and explained some regional differences between Aleppo and Latakia.

**Conclusions:**

The percentage of women who received adequate ANC content was probably higher in Latakia than in Aleppo because women in Latakia were more educated, and because services were more available, accessible, and acceptable to them.

## Introduction

Antenatal care (ANC) can detect and treat problems of pregnancy, and increase women’s chances of having a skilled attendant at birth and of seeking care promptly should complications occur [1]. Elements of ANC known to be effective include detecting and treating anaemia and preventing it [2, 3], screening and treating asymptomatic bacteriuria [4], detecting hypertensive disease of pregnancy by measuring blood pressure and analyzing urine [2] and treating severe preeclampsia and eclampsia [5]. In the Millennium Development Goal 5 (MDG5) era, (aimed at improving maternal health between 1990 and 2015), the World Health Organization (WHO) recommended four focussed ANC visits to ensure such interventions were delivered [6].

To monitor progress, countries were expected to report on the percentage of women having at least one ANC visit (1+ ANC) and four or more visits (4+ANC), but not on the adequacy of the content of care. Thus, the most studied ANC outcome is whether women attended ANC or not (coverage), and to a lesser extent, the trimester of initiation and the frequency of visits. Few studies have examined the content of care. This picture continues with the indictors for the Sustainable Development Goals (SDGs), (a subset of the heath goal being aimed at reducing maternal and neonatal mortality by 2030). WHO issued new ANC guidelines recommending initiation of ANC in the first trimester of pregnancy and minimum of 8 visits [7].

Until armed conflict started in 2011, Syria had made considerable progress in improving maternal and infant health. Maternal mortality had declined from 240 per 100,000 in 1990 to 70 per 100,000 in 2010 [8], and infant mortality had declined from 35 per 1000 live births in 1993 [9] to 16 per 1000 live births in 2005–2010 [10]. According to the Multiple Indicator Cluster Survey (MICS) conducted in 2006, the proportion of women who had received ANC was above 85%. However, the survey also revealed that some elements of ANC content were not widely adopted, and there were wide regional variations in coverage and quality of care. Apart from national surveys, few studies have investigated maternal health services utilisation in Syria, particularly content of care. The only detailed study to explore content of ANC was in the better-serviced areas of Damascus and its suburbs. It reported the percentage of women who received ultrasound examination, but not on other clinical or laboratory tests [11].

Most studies examining access to health care use frameworks that include four dimensions, each with supply and demand elements: 1) geographic accessibility (physical distance or travel time from the user to the service delivery point); 2) availability (having the right type of care available to those who need it, including hours of operation and waiting times that are acceptable to potential users, and appropriate types of service providers and materials); 3) financial accessibility, (relationship between the price of services, partly affected by their cost, and the willingness and ability of users to pay for those services considering financial protection); and 4) acceptability (responsiveness of health service providers to the social and cultural expectations of individual users and communities) [12].

Given the importance of ANC, and the evidence in the literature suggesting inadequacies in ANC content in Syria, this paper aims to address these questions:

1. How adequate is ANC content in Syria, and how did adequacy of content change from 1996 to 2006?
2. What are the supply and demand side barriers that explain the variation in adequacy of content of ANC between Aleppo and Latakia governorates?
3. To what extent does the conceptual framework used in this study (combining Kroeger’s Model, Good's Model and the Right to Health Framework) provide a means of assessing the availability, accessibility, and acceptability or quality of ANC content for women in Syria?

This paper uses the concepts of supply and demand side barriers to explain variation in the adequacy of ANC content between two governorates in Syria, using a mixed methods approach. The governorates were chosen from among the worst performing and best performing governorates (Aleppo and Latakia respectively). The study was carried out in 2009–2010, before the armed conflict in Syria started, and when all governorates were under government control. During the conflict, Aleppo has been under rebel control, while Latakia remains under government control [13].

## Material and methods

### Study setting

Syria is a lower middle-income country, and at the time of the study (2010) had a General National Income per capita of $2891 [14], and an estimated population in 2011 of 24,504,000 (14). The total fertility rate in Syria was 3.5 children per woman [15].

We researched two of the country’s 14 governorates: Aleppo, the largest governorate, where quality of ANC content, as revealed by MICS 2006 survey, was low, and Latakia, whose capital is Syria’s main port city, where quality of ANC content was high. In the rest of the paper, these governorates will be referred to as Aleppo and Latakia.

Aleppo is in northern Syria covering 1,850,000 hectares, with 8 districts and 1,453 villages. In 2011, its population was 5,927,000 (3.2 per hectare) [16]. The total fertility rate was 3.2 children per woman [15]. Latakia is in western Syria covering 230,000 hectares, with 4 districts and 432 villages [16]. In 2011, the population was 1,229,000 (5.3 per hectare). The total fertility rate was 2.2 children per woman [15].

The healthcare delivery system in Syria is provided by both the public and the private sectors. ANC services in the public sector are delivered through primary health care centres in urban and rural areas [17]. Most health providers in Syria work in both the public and the private sectors [17]. However, the Syrian Ministry of Health (MoH) regulations for providing ANC were only required for public health facilities and no regulations were imposed on the private sector.

Data on health service provision are only available for the public sector. In 2010, there were 207 public-sector health centres in Aleppo and 118 in Latakia, but the number of public-sector services per population (density) was 2.7 higher in Latakia (9.6 per 100,000) than in Aleppo (3.5 per 100,000). The densities of gynaecologists, of health centres with ultrasound, and of health centres with laboratories were also higher in Latakia than in Aleppo.

### Study design

Our conceptual framework combined Kroeger’s Model [18], Good's Model [19] and the Right to Health Framework [20] (S1 Fig). According to our framework, a woman’s choice of ANC service, and consequently the likelihood of her receiving adequate ANC content, depend on three dimensions of access: (1) availability, (2) geographical and financial accessibility and (3) acceptability of health services. Each of these dimensions is determined by a set of supply and demand side factors that enable or obstruct access to adequate ANC [21].

**Fig 1:**
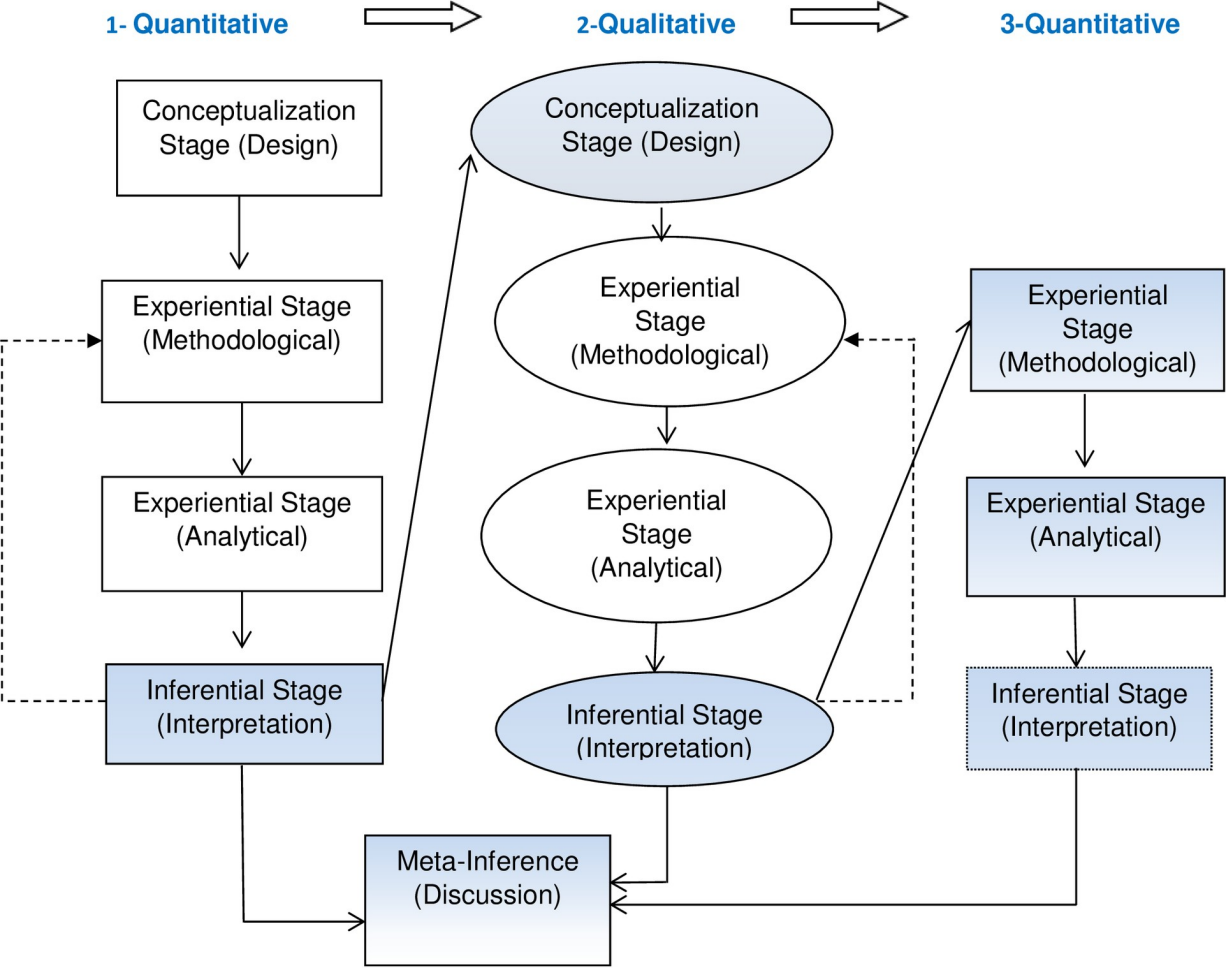
The mixed-method design used for this study, adopted from Teddlie [23].

Combining quantitative and qualitative research approaches offers a strong potential for identifying, exploring and understanding factors affecting the content of ANC [22]. We used a sequential mixed-method design (Fig 1), where quantitative research was followed by qualitative and then again by quantitative research [23, 24]. The blue shapes indicate where both methods were integrated, namely at the design, interpretation and discussion stages [20]. At the design stage, we used ‘development integration’ whereby quantitative results from the analyses of the national surveys (first phase) to examine the adequacy of ANC content and changes in this over time, informed the settings, design and data collection for the qualitative (second) phase of research. This component explored some of the supply and demand side barriers to adequate ANC content in Aleppo and Latakia governorates. The results then led to further quantitative analyses (phase three) to identify socio-economic determinants of adequacy of ANC content, which contributed to the identification of some of the supply and demand side barriers to adequate ANC. We used ‘triangulation’ and ‘complementarity’ at the meta-inference (discussion) stage, whereby all three study components complemented each other by elaborating different phenomena related to the choice of care provider, and consequently the content of ANC. The meta-inference stage was used to answer the last research question on the extent to which our conceptual framework provides a means of assessing the availability, accessibility, and acceptability or quality of ANC content in Syria.

#### Quantitative study

**Characteristics of the analysed surveys:** We analysed existing data from the 2001 Pan Arab Project for Family Health (PAPFAM) and the 2006 Multiple Indicator Cluster Survey (MICS). In these multi-stage surveys, all ever-married women aged between 15 and 49 years were interviewed and asked about ANC for their last live birth (in the five years preceding the interview for PAPFAM (4018 women) and in the last two years preceding the interview for MICS (3923 women). S1 Table describes both surveys including sampling design, administration, interviewed women, the questionnaires and the availability of outcome and independent variables. We started by analysing data at the national level, which demonstrated regional differences in coverage and content of ANC and that some governorates improved more than others over time. After the qualitative analysis (second phase of analysis), we analysed the subset of women who were ANC users in Aleppo and Latakia (third phase of analysis); the resulting sample size in phase three was 611 women (452 women in Aleppo and 159 women in Latakia) for PAPFAM and 721 women (577 women in Aleppo and 144 women in Latakia) for MICS.

**Adequacy of ANC content:** We constructed a binary composite measure for the content of ANC care based on the Syrian MoH’s recommendations, and on the availability of these variables in the survey questionnaires. The composite measure included: (1) weight measurement, (2) blood pressure measurement, (3) blood taken and (4) urine taken. We assume that taking a blood or urine sample means these were also analysed. Within this composite outcome, we refer to weight and blood-pressure measurement as clinical tests and the taking of blood and urine samples as laboratory tests assuming these samples were also analysed. ANC content was considered adequate if the woman reported having received all four elements and inadequate if any of these components were missing.

**Quantitative data analysis methods:** The percentage of ANC users who received adequate ANC content was reported in different subgroups of the population as defined by socio-demographic variables, health-related variables and variables related to previous pregnancies. Adequacy of ANC content was also compared between the surveys (PAPFAM 2001 and MICS 2006) to assess trends in each governorate.

Crude odds ratios were calculated for the association between each exposure variable and the outcome (“adequacy of ANC content”). Additionally, logistic regression was used to calculate adjusted odds ratios for the Aleppo survey. Adjusted odds ratios were not calculated for Latakia survey because the sample size (n = 159 for PAPFAM and n = 144 for MICS) was too small.

We considered age, education, area of residence, wealth quintile and parity as potential confounders. The odds ratio for each exposure variable was adjusted for age since it was unlikely to be on the causal pathway between exposures and the outcome. The other four potential confounders (education, area of residence, wealth quintile and parity) were only adjusted for when we considered they were unlikely to be on the causal pathway between the exposure and the outcome [25]. For example, for age at marriage, we adjusted for age, education and area of residence, but not socio-economic status and parity because we believed the latter variables could be on the causal pathway. (i.e. where mechanism via which age at marriage operated). As another example, for parity we only adjusted for age and education because we believed all the other factors could be on the causal pathway. The MICS 2006 included a variable for wealth quintile, but the PAPFAM 2001 survey did not, so we used principal component analysis to generate a wealth quintile variable for PAPFAM, based on the same household possessions as used in MICS.

Analyses were performed using STATA version 10, p-values and confidence intervals were calculated using robust standard errors to allow for clustered data.

#### Qualitative study

The lead author, a female Syrian PhD student, who was familiar with the culture and the setting and with previous experience in qualitative research, conducted semi-structured interviews with women, doctors, midwives and health officials and kept field notes. She introduced herself and informed the participants about the study aims and that it was part of her PhD. Each interview lasted between 30–60 minutes.

Interviewees were recruited purposefully among health providers and women in 16 private and public health facilities using an up to date list of gynaecologists practicing privately in Aleppo and Latakia from the Syrian Society of Obstetrics and Gynaecology, and a list of doctors practicing at health centres in both cities, from the MoH. We randomly selected four clinics/health centres from each list. We then called the doctor working at this clinic/health centre, explained the study and arranged an appointment to interview them. All doctors we contacted agreed to participate.

We recruited midwives who provide ANC independently (in Aleppo) with the help of health officials in Aleppo who provided us with the contact numbers of six midwives. The lead author interviewed four midwives who agreed to participate but was unable to reach the other two.

We attempted to recruit at least one woman attending ANC per clinic/health centre where the interviewed doctors/midwives worked and increased the sample until the information we received was saturated. The lead author approached the women in the waiting areas of the clinics/health centres, introduced herself, explained the study aims and asked to visit them at home. All women who were approached agreed to participate.

Women who did not attend ANC were recruited using the snowball technique, with the help of doctors and women participating in our study. We were unable to locate women who had not sought ANC in Latakia. Our experience was confirmed by doctors and women in Latakia who claimed that almost all women in Latakia attended ANC at least once, and by the high ANC coverage in MICS.

We interviewed the key health official at the health directorates located in Aleppo and Latakia, and in the central health directorate in Damascus. The lead author called them, and explained the study; all three health officials agreed to participate.

Doctors and midwives were interviewed at their clinics/health centres. Health officials were interviewed at their offices, and women were interviewed at their homes. There was no one else present during the doctors, midwives and health officials' interviews. Other people were present during most interviews with women, generally other family members or neighbours.

The lead author interviewed 8 doctors in Aleppo and 8 in Latakia, 18 women in Aleppo and 12 in Latakia, 4 midwives in Aleppo and one health official in each of Aleppo, Latakia and Damascus. There were 53 initial interviews, and 20 follow-ups, totalling 73. Sixty-eight interviews were voice-recorded, and 5 were documented in writing. Most women in Aleppo were interviewed twice, because they were shy or hesitant during the first interview, which may have influenced their willingness to disclose information about their health seeking behaviour and their perception of the content of ANC. There was an 8-week period between the first and second interviews with women. Similarly, doctors in Aleppo were interviewed twice because of their initial reluctance to share information.

The lead author explained the study to the participants using a detailed consent form, while reassuring them they would remain anonymous and could withdraw from the study at any time without giving a reason. Service providers gave written consent while women gave oral consent to participate.

We used an interview guide covering women’s practices in their current pregnancies and their perceptions of the content of ANC, with separate guides for those who did not attend ANC. Health providers were asked about their ANC practices in the public and the private sector if they had dual practices. The preliminary findings from these interviews directed the development of the guide for governorate and central level health officials about health policies and services organisation. The interview guides were pilot tested with two women and two doctors before the study started. We were unable to obtain the participants' feedback on the findings because we were unable to locate them, as the civil war started soon after data collection was completed.

**Data management and analysis of qualitative findings:** Interviews were transcribed verbatim in Arabic, and data analysis was conducted by the lead author with codes checked by a co-author. We used the framework approach, a variant of content analysis developed for policy relevant research, to analyse the data [26]. A thematic framework based on *a priori* issues derived from the study aims and newly emergent themes was constructed after familiarization with the data, and persistent views or experiences present in the data were organized. This process produced a detailed index, which helped to organize the data in a manageable manner that simplified later retrieval. Three indexes on the adequacy of content of care were produced: one for women describing the uptake of ANC tests, one for health providers describing patterns of providing ANC, and one for health officials describing structural issues that influence women’s health seeking patterns and doctors’ provision of care (S2 Fig). Relevant themes were translated into English.

We attempted to find associations between index themes and explain the findings. The following step entailed linking qualitative results with the corresponding quantitative ones, regarding each adequacy construct. It also involved identifying links among the assembled series of strategies and barriers adopted by each group and discussing them in relation to the broader context of health system organisation as well as the socio-cultural context where doctors and women lived. The last step entailed discussing the refined themes and the corresponding quantitative results under the broad components of the “Right to Health” framework (availability, geographical and financial accessibility, and acceptability or quality) of ANC services. This approach of labelling the themes simplified the integration of the final analysis, interpretation, and presentation of quantitative and qualitative results.

## Results

### Characteristics of women in 2006 by governorate

#### Demographic characteristics of women in Aleppo versus Latakia

**Fig 2:**
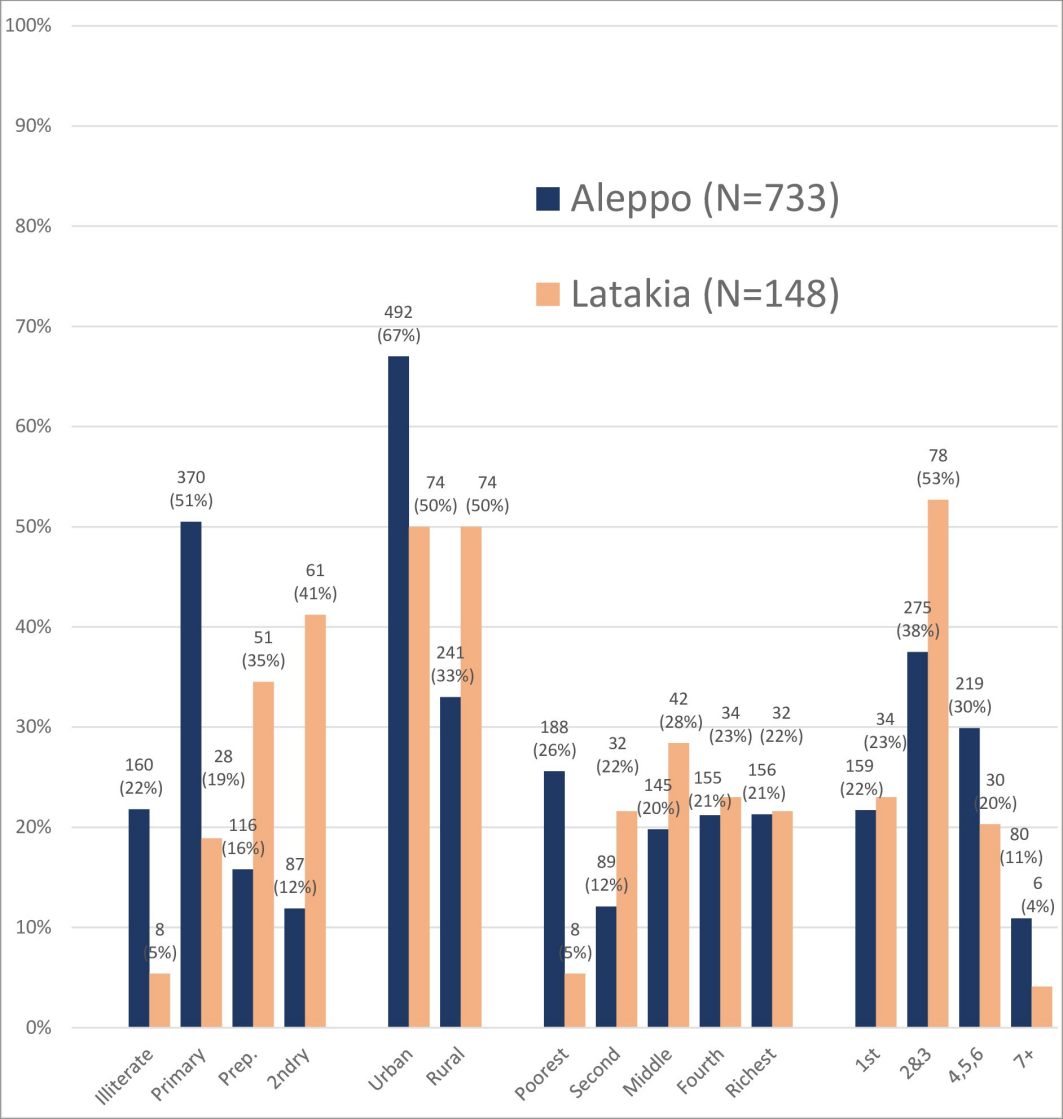
Main demographic characteristics of women by governorate, MICS 2006 survey.

Fig 2 shows that in 2006, compared to women in Aleppo, women in Latakia were more educated (41% versus 12% with secondary education, p<0.001), less poor (5.4% versus 25.7% in the poorest quintile, p<0.001), more likely to live in a rural area (50% versus 33%, p<0.001), and less likely to have high parity (4.1% versus 10.9% with seven or more children, p = 0.001).

#### Characteristics of participants in qualitative study

In Aleppo and Latakia, women’s ages ranged from 16 to 40 years. Parity was roughly similar, and in both places, many women had never worked. In Latakia, 7 out of 12 women (58%) had a university degree compared to only 3 out of 17 (18%) in Aleppo. In Aleppo, 10 out of 18 women (56%) had no ANC at all, or had ANC with a midwife, while in Latakia, none of the women had any ANC and none had ANC with a midwife.

Out of the 16 doctors in both governorates, 7 (44%) were females, and 10 (62%) worked in both the public and private sectors. All midwives practiced at private clinics, mostly in poor areas, and two had clinics in their homes.

### Quantitative findings

#### Trends in adequacy of content of ANC (phase one)

We explored the adequacy of ANC and changes over time in all of the governorates in Syria. We also compared adequacy of ANC content between Aleppo and Latakia.

The percentage of women who received adequate ANC content (weight measurement AND blood pressure measurement AND urine test AND blood test) in Syria increased substantially from 2001 to 2006 from 33% to 54%, p<0.001 (Table 1). Tartous and Latakia had the highest percentage of women who received adequate ANC content in 2004–2006, 96% and 93% respectively. Homs, Raqqa, Deir ezzor and Quneitra governorates did not experience a significant increase in the percentage of women who received adequate ANC content (Table 1).

**Table 1:** Changes over time and percentages of women who received adequate ANC content, among women who attended ANC, PAPFAM 2001, and MICS 2006, by governorate (phase one).

	PAPFAM 2001 N (%)	Total N	MICS 2006 N (%)	Total N	P-value for trend over time (Wald test)
Adequate ANC content (weight measurement+ blood pressure measurement+ blood test +urine test)					
**Syria**	957 (33.3)	2872	1811 (54.0)	3347	**P<0.001**
**Damascus**	194 (54.0)	359	187 (83.1)	225	**P<0.001**
**Rural Damascus**	200 (39.8)	503	372 (65.3)	570	**P<0.001**
**Homs**	144 (53.3)	270	174 (54.9)	317	P = 0.812
**Hama**	50 (26.0)	192	146 (62.9)	232	**P<0.001**
**Tartous**	49 (33.3)	147	155 (96.3)	161	**P<0.001**
**Idleb**	23 (19.0)	121	99 (45.8)	216	**P<0.001**
**Raqqa**	31 (24.2)	128	45 (32.1)	140	P = 0.292
**Deir Ezzor**	9 (9.9)	91	28 (13.5)	208	P = 0.683
**Hassakh**	15 (8.9)	168	59 (29.4)	201	**P<0.001**
**Suweida**	19 (24.1)	79	57 (79.2)	72	**P<0.001**
**Daraa**	23 (12.6)	183	108 (41.5)	260	**P<0.001**
**Quneitra**	4 (20.0)	20	5 (20.8)	24	-
**Latakia**	109 (68.6)	159	134 (93.1)	144	**P<0.001**
**Aleppo**	87 (19.3)	452	242 (41.9)	577	**P<0.001**
P-value comparing (Aleppo and Latakia governorates)	**P<0.001**		**P<0.001**		
**A-Weight measurement**					
Latakia	141(88.7)	159	141 (97.9)	144	**P = 0.023**
Aleppo	310 (68.6)	452	434 (75.2)	577	P = 0.086
P-value comparing governorates	**P<0.001**		**P<0.001**		
**B-Blood pressure measurement**					
Latakia	151 (95.0)	159	139 (96.5)	144	P = 0.547
Aleppo	374 (82.7)	452	503 (87.2)	577	P = 0.173
P-value comparing governorates	**P = 0.001**		**P = 0.001**		
**C-Blood test**					
Latakia	124 (78.0)	159	135 (93.8)	144	**P = 0.004**
Aleppo	152 (33.6)	452	330 (57.2)	577	**P<0.001**
P-value comparing governorates	**P<0.001**		**P<0.001**		
**D-Urine test**					
Latakia	130 (81.8)	159	135 (93.8)	144	**P = 0.016**
Aleppo	153 (33.9)	452	275 (47.7)	577	**P<0.001**
P-value comparing governorates	**P<0.001**		**P<0.001**		

The results in this phase generated interest in two particular governorates that experienced a significant increase in adequacy of ANC content between 1996 and 2006 (Table 1). The first was Aleppo where the quality of ANC content remained low despite the significant improvement between the two time periods. The second was Latakia, where the quality of ANC was low initially but showed noticeable improvements over time such that the quality of ANC became comparable to that in Damascus, the capital. Therefore, besides the capital, Damascus, where quality of care is expected to be high, among the governorates whose adequacy of ANC content remained low in 2004–2006, Aleppo was the largest and most important, and among the few governorates that had high adequacy of ANC content in 2004–2006, Latakia was also among the largest and the most important.

More women in Latakia received adequate ANC content in 2001 than in Aleppo (59% versus 19%) but there was an upward trend over time in both governorates (from 59% to 93% in Latakia and from 19% to 42% in Aleppo).

In both the 2001 PAPFAM and the 2006 MICS survey, the percentage of women who received each component of ANC was higher in Latakia than in Aleppo. In both governorates and at both time periods, more women had clinical tests than laboratory tests. The percentage of women tested increased significantly over time in both governorates apart from blood pressure measurement in Latakia (Table 1).

#### Determinants of adequate ANC content (phase 3)

The analysis stratified by governorate revealed that more women in Latakia received adequate ANC content than women in Aleppo even when they shared the same socio-demographic characteristics (S2 Table). For instance, 64% of the most educated women and 56% of the richest women in Aleppo received adequate ANC content compared to 100% of the most educated and richest women in Latakia.

The multivariable analysis of MICS data from Aleppo showed that lower education increased the odds of not receiving adequate ANC content, while seeking care at a public health facility decreased the odds, although the decrease was only of borderline significance (Table 2). Woman’s age, wealth, area of residence, age at marriage, and whether the pregnancy was wanted were not associated with receiving adequate ANC content. In the PAPFAM survey, the only variable that increased the odds of not receiving adequate ANC content was having no current health complications. Woman’s employment status, habit of reading the newspaper, suffering from long-term illness, having had an abortion, husband’s education level and employment status and having a problem with the unavailability of a female health provider, had no influence on adequacy of ANC content (Table 2).

**Table 2:** Determinants of not receiving adequate ANC content in Aleppo, among women who attended ANC in MICS 2006, and PAPFAM 2001 (Phase 3).

**MICS 2006 (N = 577)**	**Not receiving adequate ANC content** **335 (58.1%)**	**P-value**	**Unadjusted Odds Ratio (95% CI)**	**Adjusted Odds Ratio (95% CI)**	**Covariates adjusted for†**	**P-value**
**Woman's age**						
15–19 years (N = 50)	26 (52.0)	P = 0.129	1.00		None	
20–24 years (N = 163)	91 (55.8)		1.17 (0.60–2.25)			
25–29 years (N = 160)	98 (61.3)		1.46 (0.75–2.83)			
30–34 years (N = 95)	46 (48.4)		0.87 (0.43–1.76)			
35–30 years (N = 80)	53 (66.3)		1.81 (0.83–3.96)			
> 40 years (N = 29)	21 (72.4)		2.42 (0.85–6.91)			
**Woman’s education**						
Secondary or higher (N = 81)	29 (35.8)	**P<0.001**	1.00	1.00	1	**P<0.001**
Preparatory (N = 101)	49 (48.5)		1.69 (0.94–3.03)	1.86 (1.00–3.45)		
Primary (N = 294)	175 (59.5)		2.64 (1.54–4.51)	2.85 (1.62–5.01)		
Illiterate (N = 101)	82 (81.2)		7.74 (4.14–14.47)	7.88 (4.07–15.25)		
**Residence**						
Urban (N = 415)	220 (53.0)	**P<0.001**	1.00	1.00	1,2	P = 0.079
Rural (N = 162)	115 (71.0)		2.17 (1.44–3.27)	1.52 (0.95–2.42)		
**SES quintiles**						
Richest (N = 144)	64 (44.4)	**P<0.001**	1.00	1.00	1,2	P = 0.091
Fourth (N = 129)	66 (51.2)		1.31 (0.79–2.18)	1.02 (0.59–1.75)		
Middle (N = 120)	74 (61.7)		2.01 (1.11–3.64)	1.60 (0.89–2.88)		
Second (N = 63)	41 (65.1)		2.33 (1.21–4.49)	1.74 (0.89–3.40)		
Poorest (N = 121)	90 (74.4)		3.63 (2.10–6.27)	2.01 (1.05–3.84)		
**Parity**						
First child (N = 136)	65 (47.8)	**P = 0.003**	1.00	1.00	1,2	P = 0.097
Second-third (N = 226)	128 (56.6)		1.43 (0.91–2.25)	1.44 (0.89–2.33)		
Fourth, fifth, sixth (N = 164)	100 (61.0)		1.71 (1.04–2.81)	1.56 (0.84–2.91)		
Seventh or more (N = 51)	42 (82.4)		5.10 (2.12–12.27)	3.63 (1.30–10.13)		
**Age at marriage**						
>25 (N = 38)	15 (39.5)	**P = 0.014**	1.00	1.00	1,2,3	P = 0.090
22–25 (N = 126)	62 (49.2)		1.49 (0.71–3.09)	1.65 (0.78–3.52)		
18–21 (N = 252)	156 (61.9)		2.49 (1.21–5.13)	2.39 (1.13–5.07)		
<17 (N = 161)	102 (63.4)		2.65 (1.23–5.73)	2.60 (1.13–5.96)		
**Desired pregnancy**						
Yes (N = 488)	285 (58.4)	P = 0.741	1.00	1.00	1,2,3,4,5	P = 0.052
No (N = 89)	50 (56.2)		0.91 (0.53–1.56)	0.58 (0.33–1.00)		
Type of health facility*						
Private (N = 420)	246 (58.6)	P = 0.323	1.00	1.00	1,2,3,4,5	**P = 0.016**
Public (N = 120)	62 (51.7)		0.76 (0.43–1.32)	0.48 (0.27–0.88)		
**PAPFAM 2001 (N = 452)**	**Not receiving adequate ANC content** **365 (80.7%)**	**P-value**	**Unadjusted Odds Ratio (95% CI)**	**Adjusted Odds Ratio (95% CI)**	**Covariates adjusted for**†	**P-value**
**Working status of woman**						
Currently working or worked in the past (N = 59)	45 (76.3)	P = 0.383	1.00	1.00	1,2	P = 0.799
Never worked (N = 393)	320 (81.4)		1.36 (0.68–2.74)	1.10 (0.52–2.36)		
**Read newspapers**						
Yes (N = 116)	86 (74.1)	**P = 0.047**	1.00	1.00	1,2,3,4	P = 0.840
No (N = 336)	279 (83.0)		1.71 (1.01–2.89)	1.07 (0.56–2.04)		
**Health complications**						
At least one symptom (N = 119)	80 (67.2)	**P<0.001**	1.00	1.00	1,2,3,4,5	**P<0.001**
No complications (N = 333)	285 (85.6)		2.89 (1.69–4.96)	3.36 (1.86–6.08)		
**Long-term illness**						
Yes (N = 45)	32 (71.1)	P = 0.090	1.00	1.00	1,2,3,4	P = 0.165
No (N = 407)	333 (81.8)		1.83 (0.91–3.67)	1.70 (0.76–3.81)		
**Previous abortion**						
Yes (N = 63)	49 (77.8)	P = 0.477	1.00	1.00	1,2,3,4	P = 0.296
No (N = 389)	316 (81.2)		1.24 (0.69–2.22)	1.38 (0.75–2.54)		
**Husband’s age**						
>41 (N = 84)	69 (82.1)	P = 0.710	1.00	NA		
34–41 (N = 164)	129 (78.7)		0.80 (0.42–1.55)			
26–33 (N = 156)	126 (80.8)		0.91 (0.46–1.80)			
18–25 (N = 48)	41 (85.4)		1.27 (0.49–3.32)			
**Husband’s education**						
Secondary or higher (N = 99)	74 (74.8)	P = 0.315	1.00	1.00	1	P = 0.481
Preparatory (N = 71)	60 (84.5)		1.84 (0.88–3.88)	1.75 (0.83–3.69)		
Primary (N = 177)	146 (82.5)		1.59 (0.83–3.05)	1.43 (0.73–2.79)		
Illiterate (N = 105)	85 (81.0)		1.44 (0.78–2.65)	1.33 (0.71–2.51)		
**Husband’s work status**						
Does not work in agriculture (N = 413)	331 (80.2)	P = 0.240	1.00	1.00	1	P = 0.277
Jobless or works in agriculture (N = 39)	34 (87.2)		1.68 (0.71–4.02)	1.62 (0.68–3.87)		
**No female health provider**						
No problem (N = 268)	206 (76.9)	**P = 0.026**	1.00	1.00	1,2,3,4,5	P = 0.069
Problem (N = 184)	159 (86.4)		1.91 (1.08–3.38)	1.76 (0.94–3.27)		

†Odds Ratio (OR) adjusted for (1) woman’s age, (2) education, (3) area of residence, (4) socio-economic status and (5) parity.

* 20 women who attended ANC at both sectors and 17 women who did not have their ANC at health facility were dropped from the analysis

#### Public and private health sector differences (phase 3)

We also investigated how adequacy of ANC content differed between the public and private health sectors to identify the supply and demand side barriers to adequate ANC content. Exploring public and private health sector differences in 2006, (S3 Table) revealed that in Latakia the private sector offered more adequate ANC content than the public sector (96% vs 67%), while in Aleppo there was no difference between the private and public sectors (41% vs 48% respectively). However, further multivariable analysis in Aleppo showed that the odds of receiving non-adequate clinical and laboratory tests were lower among women attending the public sector than women attending the private sector.

The MICS survey did not report data on the timing and frequency of ANC. Among women identified in the 2001 PAPFAM survey as having started care early and having made at least four ANC visits (S3 Table), the proportion who received adequate ANC content was similar in women who attended the public and the private sector in Latakia (72.7% vs 71.4% respectively) but was much higher in women who attended the private sector in Aleppo (31.7% for women attending the private sector vs 14.8% for women attending the public sector). Even among women who started care early and made at least four ANC visits in Aleppo, only 41% of women who attended the public sector and 73% of women who attended the private sector received adequate clinical tests. Similarly, only 22% of women who attended the public sector and 36% of women who attended the private sector in Aleppo received adequate laboratory tests (S3 Table).

#### Qualitative findings: Barriers to provision and reception of adequate ANC content (phase 2)

Our quantitative results showed that fewer women received laboratory tests than clinical tests, particularly in Aleppo. The qualitative findings revealed three themes that could explain some of the supply side and demand side barriers to adequacy of care and why fewer women in Aleppo had laboratory tests, leading to a lower adequacy of ANC content compared to Latakia.

#### Organizational issues at the health policy level

The relative scarcity of health facilities and human resources in Aleppo made regular supervision to monitor adherence to ANC regulations harder than in Latakia, where the density of health facilities and human resources was higher. Additionally, Aleppo’s large population and geographical size, and its larger population per health service, made it more difficult for staff working at public health centers to follow women up. A health official in Aleppo explained:

*“We have 12 areas and 264 health centers in Aleppo*. *Latakia is much smaller*, *the need in Aleppo is much bigger*, *and there is a deficiency in providing regular supervision because there are no fieldworkers to do this*.*” (Health official 2)*

The health official also mentioned demotivated staff when asked how he ensured staff and doctors in Aleppo follow regulations:

*“The problem is that they have long working hours and no motivation at all*.*” (Health official 2*)

*The health official in Damascus complained*:

*“There is misdistribution of resources*. *Aleppo is big*, *its size is equal to two governorates*, *and health officials treat it as a small governorate*.*” (Health official 1*)

The relatively low resource allocation in Aleppo resulted in fewer doctors providing ANC per woman of reproductive age than in Latakia, which in turn led to lower competition among doctors working at public health centers. Consequently, doctors in Aleppo were less apprehensive about losing their jobs, with less incentive to follow MoH regulations as strictly as doctors in Latakia. One health official elaborated:

*“Doctors in Aleppo don’t follow the health center policies*. *There is no communication between the doctor and the midwife*. *In Latakia*, *there are a lot of doctors working at health centers and doctors worry about losing their jobs so they are more committed to policies*.*” (Health official 1*)

The limited resources in Aleppo made it more difficult to motivate staff. The same health official claimed that doctors working at health centers in Aleppo changed frequently, especially in rural areas, because they were dissatisfied with working conditions. This made it harder for the doctors to develop strong relationships with health center staff and to communicate effectively with them. Interviews showed that doctors working in the public sector in Aleppo were often unaware what laboratory tests were available locally.

One common issue at several health centers in Aleppo, but not in Latakia, was the lack of equipment for basic blood and urine tests. When asked why many laboratories are not equipped to perform blood and urine analysis, one health official claimed:

*“Economically equipping health centers with laboratory equipment is not correct because you cannot equip a health center that does not have a high work load*. *This equipment will be damaged after a while*.*” (Health official 3*)

Since Aleppo is less densely populated than Latakia (3.2 population per hectare versus 5.3), and has more remote areas that are further from urban centers, this is a possible explanation. According to health officials, inadequacies in physical resources and mismanagement at the district and health center levels could have contributed to the lack of equipment, exacerbated by inadequacies in the distribution of human resources, the failure to train local staff to conduct basic tests, the lack of clear managerial roles, and the unwillingness of lab technicians to work in remote rural areas due to poor transportation and incentives.

#### Doctors’ selective prescription of ANC tests

Not all interviewed doctors requested the routine ANC laboratory tests recommended by the MoH, especially in Aleppo. One reason for selectively prescribing ANC tests was the lack of access to certain tests at the health centers where doctors worked. A female doctor, who practiced at a health center and a private clinic in a village in Aleppo, said:

“*We do not have an equipped laboratory at the health center*, *and if I ask women to do the tests outside the center they won’t go*. *People who come to the health center are poor and we cannot offer them anything*.*” (Doctor 7*, *Aleppo)*

Sometimes, doctors who provided care in rural areas said they refrained from referring their poor patients to other public health centers that had tests because these centers were far from where women lived and they wanted to avoid imposing additional transportation expenses. A male doctor who practiced at a public health center and a private clinic at a village in Aleppo explained:

*“The closest health center that provides the tests for free is far in Manbij center*, *which is half an hour by car or bus” (Doctor 8*, *Aleppo*)

Many doctors working in the private sector in Aleppo said they sympathized with their poor patients’ financial situation. Therefore, they tried to minimize their patients’ medical expenses by only asking for laboratory tests they thought were indispensable, or by referring women to public hospitals where tests were free.

Some doctors felt that asking for too many laboratory tests might discourage women from undergoing those tests they deemed essential. A female doctor, who worked at a health center and a private clinic in Aleppo, said:

*“In my clinic there are patients who cannot afford the tests*. *It depends on the patient*, *if there are less necessary tests*, *I do not ask for them because I am worried that she might cancel all the tests and not do any of them*.*” (Doctor 2*, *Aleppo*)

A few doctors in Aleppo explained that if women had health insurance, doctors would feel comfortable asking them to do routine ANC laboratory tests and would not give in to women’s unwillingness to have the tests for reasons of cost. When dealing with poor or hesitant patients, doctors in Latakia followed a different strategy by referring women to public health centers where the tests are free.

Some doctors in Aleppo believed that they would be perceived as incompetent and incapable of diagnosing illnesses if they asked women for tests. One doctor practicing in a village in Aleppo narrated:

*“Many doctors prescribe medication based on the clinical examination and if the woman does not improve they ask her to do the test*. *If we ask for the tests*, *sometimes they accuse us of inability to diagnose them properly*. *You know*, *women in these areas do not have high awareness*.*” (Doctor 8*, *Aleppo*)

Several doctors working in the private sector in Aleppo articulated both their worries about independent midwives providing ANC for lower fees and their concerns about midwives’ limited competence to provide ANC. Doctors expressed their frustration about the lack of regulation against this recent development, which they believed was causing them lose patients to midwives.

For example, a doctor who worked at a private hospital and had his own private clinic said:

*“Midwives are providing ANC for many pregnant women in Aleppo and their clinics should be shut down*. *They do not have enough experience*.*” (Doctor 3*, *Aleppo*)

Similarly, a female doctor who practiced at her own clinic in Aleppo expressed her aggravation about midwives:

*“Many women go to midwives because they are cheaper*. *Their husbands take them to those midwives*. *They should stop midwives from providing ANC*. *How could a midwife who studied for three years compete with a doctor who studied for twelve years*? *It is just not fair*.*” (Doctor 4*, *Aleppo*)

These examples suggested that doctors felt threatened by midwives taking over the care of pregnant women. Therefore, doctors might have adopted some of the above-mentioned strategies to avoid losing their patients, by prioritizing the laboratory tests they asked women to do to minimize costs and inconveniences of seeking care at public health facilities. This was not mentioned as a problem in Latakia.

In summary, doctors practicing in Aleppo discussed factors such their limited access to free ANC laboratory tests locally, the need to limit the cost of tests to private sector clients, and perceived competition from independent midwives on both cost and competency while these factors were not brought up by doctors in Latakia.

#### Women’s selective uptake of ANC tests

Women in Aleppo commonly reported making their own decisions on which laboratory tests and medications to take, sometimes irrespective of their doctors’ advice. This practice was mainly related to how women perceived their pregnancy and the possible risks that could affect them. This could result both in women not taking up laboratory tests prescribed by doctors, and in undertaking tests not prescribed by their doctors.

Some women who had normal test results in the current pregnancy or in previous ones, or who had not experienced complications in the current or previous pregnancies were not inclined to have or repeat tests. For instance, a 29-year old woman who was pregnant for the sixth time and who attended care with a midwife, explained:

*“I do not want to repeat the tests in each pregnancy*, *I did the tests in the first pregnancy*, *and I had nothing*.*” (Woman 14*, *Aleppo*)

On the other hand, a 24-year old woman who was pregnant for the second time, and attended ANC with a private doctor, explained:

*“I heard that toxoplasmosis can kill the baby and I told my doctor about toxoplasmosis*. *He told me you don’t have to do it*, *this would be foolish because it costs 2000 SP [40 USD]*. *I am planning to do the test even if my doctor did not ask for it*.*” (Woman 18*, *Aleppo*)

Additionally, women doubted the accuracy of tests provided at the labs at public health centers and complained about the long waiting hours at those centers. One of the midwives explained:

*“I send my patients who cannot afford tests to the public hospital but if you don’t know anybody there they don’t respond immediately*, *and you have to wait*. *It is a big problem*. *The woman goes and spends all day to get a test*.*” (Midwife 3*, *Aleppo*)

Sometimes women were not aware that health centers provided the basic ANC laboratory tests. This phenomenon was more common in Aleppo. One woman complained:

*“The staff did not mention that I could do some of the tests at the health center*.*” (Woman 8*, *Aleppo*)

A commonly used phrase by many women, particularly in Aleppo, was: “*The ultrasound reveals everything*.” They explained that they only felt reassured once they were examined by ultrasound and the doctor or midwife confirmed that the baby was healthy. A woman who sought care with a midwife explained:

“*The echo [ultrasound] shows you everything*, *it reassures you that you and your baby are ok*, *and shows you new things each time*. *One time she showed me that the movement of my baby is a bit slow and asked me to increase my fluids’ intake to increase his movement*.*” (Woman 15*, *Aleppo*)

The perceived reassurance provided by ultrasound may have discouraged some women from taking up prescribed blood and urine tests, including primiparous women. Women in Latakia exhibited greater awareness than women in Aleppo that ultrasound complements but does not replace laboratory tests.

Doctors’ testimonies indicated that women’s preference for ultrasound over laboratory test and the unavailability of the ultrasound at some health centers may discourage some women from attending the health centers. A female doctor in a village in Aleppo said:

“*Women who attend care at the health center are not convinced that they should do the tests because there is no ultrasound*. *They will only get convinced when an ultrasound becomes available*. *They won’t listen to you when you tell them how important the tests are*, *they never come back*.*” (Doctor 7*, *Aleppo*)

Some women in Aleppo reported not doing the tests because their midwives or doctors did not ask for them. A woman narrated:

*“If the doctor asks I will do them and I won’t care about the expenses but he did not*.*” (Woman 18*, *Aleppo*)

## Discussion

In Syria in 2004–6, 85% of women had at least one ANC visit, however our analyses showed that only 54% of women who attended ANC, had adequate content of ANC, and that levels of adequate content varied dramatically by governorate (42% in Aleppo compared to 93% in Latakia). The main reason for the low adequacy was the absence of laboratory tests. Several structural, provider, and women’s factors served as barriers to women receiving adequate content of ANC, which could explain the discrepancy in adequacy of ANC content between Aleppo and Latakia. These are discussed under the three key components of the “Right to Health” framework.

### Availability

Information from the MoH indicated that Latakia had more public health centres with equipped laboratories, ultrasound machines and gynaecologists per woman at reproductive age, and a better supervision system than Aleppo. It was also more densely populated with fewer remote areas. Many doctors working at public health centers in Aleppo reported not asking women to do the laboratory tests recommended by MoH because they were unavailable at the health centers and it was inconvenient to obtain them elsewhere. In line with our findings, shortages in medical supplies, problems between health care providers and administration, and inadequate reimbursement for staff working at public health centers also contributed to the low quality of reproductive health centers in neighboring Jordan [27].

However, the lack of laboratories does not fully explain why the recommended blood and urine tests were not carried out, since such simple tests could be performed using Haemocue’s and dipsticks (although these commodities would still have to be provided by the MoH or be available at low cost in local pharmacies).

The lack of information on services provided by health facilities acts as a demand side barrier [21]. When doctors or women do not know which services are offered by health facilities, they are less likely to use them. This was reported as preventing some women in both governorates from taking up laboratory tests at public health facilities.

Currently, availability of health services has been enormously disrupted by the destruction of health facilities and targeting of health professionals, particularly in Aleppo. Furthermore, health facilities that are not destroyed have additional challenges to accommodate the additional needs of internally displaced populations (IDPs).

### Accessibility

#### A-Geographical accessibility

Geographical accessibility is mainly determined by travel time, which is in turn influenced by urban-rural residence, topography, distance to health facility, and transport infrastructure and mode [12]. The quantitative analysis of MICS 2006 in both governorates showed that there were no significant differences in adequacy of ANC content between women living in urban and rural areas. However, the analysis showed that more women in Latakia received adequate ANC content even when they shared the same urban or rural residence status as women in Aleppo. We could not examine the effect of travel time on adequacy of ANC content as women were only asked about distance to the last facility attended for ANC and not distance to the closest facility capable of providing laboratory tests or even distance to the closest facility. The effect of distance on access to health services becomes stronger when combined with the lack of public transportation and with poor infrastructure [28], which was the case in Aleppo. This might have imposed additional costs on women who may have wished to have the tests done at another health facility where lab tests were available. Although not brought up by women, we suspect that, as in similar settings, the requirement for women to be accompanied by male companion in the more traditional Aleppo governorate may have been an additional barrier to travelling further for laboratory tests, especially as many public health services had limited hours, coinciding with when male family members are usually working [29, 30].

The conflict has reduced the numbers of functioning facilities, and combined with the lack of security, may have caused additional challenges to reach health services, even for women who originally had easy access to health services before the eruption of the conflict.

#### B-Financial accessibility or affordability

There was little variability in the adequacy of ANC content in Latakia, with high levels throughout. The quantitative analysis in Aleppo, where adequacy was lower, demonstrated that wealth and husband’s employment did not influence adequacy of ANC content.

Multivariable analyses showed that after controlling for potential confounders, adequacy of ANC content was slightly higher in the public sector than it was in the private sector. Our qualitative findings also showed that women attending the private sector do not always receive adequate ANC content. This finding is not surprising. A study by Powell-Jackson and colleagues [31] comparing content of care in the private and public sector across many countries, found they were not dissimilar. Moreover, in the Middle East Region, the adequacy of content was more variable in the private sector by wealth than in the public sector. In Syria, ANC guidelines issued by the MoH apply only to the public sector, therefore, private doctors are not obliged to comply with such guidelines in their private practice and those who do not practice in the public sector as well, do not receive training. Also, we were unable to limit this analysis to women who started care early and who made at least 4 visits for the more recent survey (MICS 2006), because MICS 2006 did not include variables on timing and frequency of care. Consequently, we were unable to explore if low adequacy of ANC content is a result of the health system performance or rather the result of women’s lack of attendance.

Some private doctors in Aleppo reported not prescribing laboratory tests for women because they assumed many women could not afford them, given the lack of a proper health insurance system. Doctors’ justification may indicate marginalization of poorer women and a paternalistic approach, as taking the decision on behalf of the woman, without consultation, denies poorer women the option to decide for themselves. The cost of blood and urine ANC tests is only 300 SP, equivalent to $6. This is unlikely to be unaffordable even for poor women in Syria, where the GDP per capita in 2010 was $2891. Doctors were also remiss in completing clinical tests, which only cost provider time. Additionally, the qualitative results showed that many women were willing to undertake tests prescribed by their doctors. Doctors’ consideration for their poor patients in the current study may have resulted in controlling women’s access to tests by not prescribing them. This would put a greater burden on women to find out which tests they need. Alternatively, they may have been using cost and women’s poverty as rationalizations for poor practice, although to our knowledge, this was not reported by other studies.

Even when services at public health facilities were free at the point of use, the absence of laboratory tests, particularly in Aleppo, meant additional funds were needed to take these tests elsewhere. If laboratory tests could not be taken the same day in the same town, women faced additional travel costs, especially in Aleppo with its large rural distances and poor infrastructure, presenting a major barrier for the poorest groups [29]. There is evidence from Africa that costs of services may not represent a major barrier even for the poor, if quality of services is improved [32].

Women’s employment status did not independently influence the outcome measure. A previous study in Damascus [9] showed no influence of women’s employment on ANC seeking patterns but did not examine content of ANC. Generally, in low-income countries or traditional Muslim cultures, most women’s employment is induced by poverty and is unlikely to increase service use [33].

The current Syrian conflict has caused living conditions to deteriorate markedly [34]. As a result, many women have considerably reduced resources, and may well prioritize other basic needs over health care access.

### Acceptability

According to the conceptual model, two issues determine acceptability of health services on the supply side: staff’s interpersonal skills, including their ability to win their patients’ trust, and their gender. The latter is only tangentially related to adequacy of content, via a woman’s reluctance to be attended by or to comply with care from a provider with an inappropriate gender. The surveys did not assess staff interpersonal skills or the level of trust that women had in health providers. The qualitative findings suggested that women's lack of trust in services provided at the public sector led them to sometimes ignore advice.

Quantitative results showed that there was no difference in adequacy of ANC content between women who had a problem with the unavailability of a female health provider and those who did not think it was a major issue. Although the adjusted results were not statistically significant, there was an elevated odds ratio for not receiving adequate ANC content among women who reported a problem with the unavailability of a female provider. Moreover, women were probably referring to the doctor providing ANC and not the lab technician conducting the tests.

The majority of women in the qualitative study in both governorates valued ultrasound examination and reported that they would not seek care at a health facility if ultrasound examination was not provided. Perceptions of the value of ultrasound examination in this study match the results of a previous qualitative study in Damascus [35] where all women perceived the ultrasound as the most precise diagnostic tool during pregnancy. These results also correspond with Turan’s study on the quality of hospital-based ANC in Istanbul, demonstrating that extensive use of technology was at the expense of basic components of ANC [36]. Another study in Turkey [37] found that 18% of 295 women who received ten or more ANC ultrasound examinations did not receive blood and urine tests.

Currently, the large numbers of IDPs may have affected acceptability of health services. Women who are internally displaced may have been obliged to seek care at different health facilities than the ones they are used to. Additionally, those health facilities are more likely to be overburdened and understaffed, and some populations may distrust government services.

### Interaction between barriers

Access to care results from interactions between health provider factors and patient factors, as discussed by Balabanova and colleagues [38] in the context of complex health systems. They argue that health providers offer services based on established norms, the time available, financial gain, and their perception of the patient’s financial status. Consequently, patients develop a variety of coping strategies, which are influenced by choices previously made by people in their social circle, a phenomenon termed as “adaptive expectations” by economists [39] cited in [38].

Adaptive expectations were manifested on several occasions in this study. For example, the perception of quality, an important determinant of service use in our study, was not only shaped by women’s characteristics and experiences, but also by health providers’ behaviors, which in turn was determined by the structure of the health system. Another example is the provision of ANC by independent private midwives in Aleppo, which despite being illegal attracted many women for financial and cultural reasons. Accordingly, and out of fear of losing patients, doctors tried to please their poorer patients by asking for fewer ANC laboratory tests, or by relying on clinical examination alone; this in turn might have reduced other women’s willingness to take laboratory tests requested by their doctors. Similarly, the unavailability of ANC laboratory tests at many public health centers in Aleppo, resulting from mismanagement at the health center level or the central health directorate level, obliged doctors to forego asking their patients to take the tests, or required asking them to take the tests at other public health centers or private laboratories. Consequently, some women never returned to these doctors because they did not have test results to share.

## Limitations

Our quantitative data were obtained from secondary sources, but both PAPFAM and MICS surveys were nationally representative surveys, which used similar sampling strategies, with support from large international survey programmes. They both asked similar questions using standardised questionnaire, however data were limited. The MICS 2006 survey lacked information about the timing and the number of ANC visits, two variables of particular interest in the present study, and none of the surveys included questions related to cost of care, cost of laboratory tests, or household income, which would have allowed estimation of the affordability of ANC. Recall bias might have affected some responses, especially women recalling their last delivery up to five years before the PAPFAM survey was conducted and up to two years before MICS. Consequently, respondents might not have been able to remember details about their ANC, such as the laboratory tests they took.

Because of the large number of comparisons made, there is a risk that some of the associations identified in this study are attributable to chance.

Qualitative interviews of the women at home made them feel more at ease, since they were at a familiar setting. However, interviews were often interrupted, with other family members participating occasionally. This may have discomforted women and altered their answers. To avoid this kind of bias, we interviewed women in Aleppo for a second time to provide more opportunities to interview them alone, and for a longer period, in order to help build rapport, and encourage the women to feel comfortable sharing information about their ANC practices.

## Recommendations and conclusions

This study showed that despite the high ANC coverage in Syria before the conflict and the significant improvement of adequacy of ANC content between 1996 and 2006, many women did not receive adequate ANC content, particularly laboratory tests, and there were wide regional variations in adequacy of ANC content. The quantitative analyses showed that there were some important socio-demographic determinants for not receiving adequate ANC content such as lower education level and not experiencing current health problems. However, the quantitative analyses comparing Aleppo and Latakia governorates also revealed that that more women in Latakia received adequate ANC content than women in Aleppo even when they shared the same socio-demographic characteristics. This was further explained by our qualitative findings, which highlighted important elements that contributed to such regional variations and these included organizational issues at the health system level, which influenced doctors’ practices leading them to selectively prescribe ANC tests (on the supply side) and consequently resulting in women’s selective uptake of ANC tests (on the demand side). Despite the importance of the effect of some of the women’s socio-demographic characteristics on adequacy of care, it appears that ANC services were more available, accessible and acceptable to women in Latakia than women in Aleppo.

The conceptual framework used in this study (combining Kroeger’s Model, Good's Model and the Right to Health Framework) in addition to using a mixed-methods approach, provided a comprehensive means that enabled us to understand access to adequate ANC content in two governorates in Syria by addressing supply and demand side barriers in relation to different dimensions of access. To this end, we interviewed all concerned stakeholders including women, providers and health officials involved in health care access to understand the role of contextual factors in mediating health care seeking and health care provision processes.

Our results suggested that to achieve adequate ANC, a number of issues should be addressed in parallel. Socioeconomic improvement would result in increasing the availability of ANC tests (on the supply side), and in improving the educational level of women and their socio-economic status (on the demand side), increasing women’s demand for ANC services including ANC tests. At the same time, a number of policy and structural issues need to be addressed too, as indicated by health officials. The current health system is fragmented and pluralistic. ANC is provided in both of the private and public health sectors. Some of the reported problems can be addressed within the current fragmented system such as expanding ANC regulations to the private sector, while ensuring better supervision to make sure that all doctors abide by ANC regulations regardless of the financial status of their patients. Another example would be distribution of resources according to the governorates’ size, to avoid shortages in lab equipment and supplies in larger governorates such as Aleppo. Improvement in communication is also important and can be achieved by training all relevant stakeholders. Although such recommendations may be useful, they are unlikely to be able to address the underlying problem with services that are fragmented by separate vertical programs, which often results in lack of access to care, lack of continuity and services that fail to meet the women’s needs and expectations. Additionally, inequity may persist due to access barriers and low quality of services [40]. We believe that a more comprehensive and effective approach is needed to address the current fragmentation in the health system. Efforts aimed at strengthening ANC services should focus on commitment to universal health coverage (UHC) and the introduction of a strong primary health care system, which resulted in better maternal and child health outcomes in various low and middle-income countries (LMICs) countries dealing with political and economic constraints [41]. Such efforts would need to address the cultural and financial barriers to access care, improve the quality of services to ensure continuity of care and integrate programs to maximize the contact between women and health services. Investment in a primary health care system by introducing integrated community based health care, while drawing on previous lessons of integration initiatives from LMICs [42], would not only improve maternal and child health but it will likely contribute to improving the health status of the Syrian population [43].

The Syrian war, which started after fieldwork was completed, caused considerable destruction across the country, including in Aleppo and Latakia. Many health facilities were destroyed, including those visited in Aleppo for our fieldwork. Accessibility has been compromised by the destruction of infrastructure, lack of transportation, targeting of health professionals, and the continuous fighting, all of which pose additional dangers to women. As a result, coverage of ANC in Syria has decreased from 88% in 2009 to 62% in 2013 [44]. This makes it very likely that for women remaining in Syria, the adequacy of the content of care has deteriorated even further. Although our recommendations were shared with health officials, and may still be valid in areas that were not destroyed, even health facilities in such areas face a heavy burden of accommodating IDPs, of whom, over 50% are children and women [44]. These challenges, which are augmented by the deficiency of resources and the split of the public health responsibility between opposing forces [44], make it difficult to implement any recommendations. We believe that some of our recommendations can be implemented in governmental controlled areas, but we have little information on areas that are currently under opposition control. Our hope is that our findings will be relevant again once the conflict has ended and health services are rebuilt. The existing culpabilities in the health system organization that resulted in low adequacy of ANC care content should be avoided when rebuilding the health infrastructure. Meanwhile, reconstructions efforts cannot begin as long there are still areas of conflict in the country. Furthermore, rebuilding the economy would be crucial before such efforts can even start. For other countries with similar health systems, income levels and contextual factors and challenges, such as Jordan and Egypt, where health systems are highly dependent on the private sector for ANC provision and where adequacy of ANC content remains low [45], some of our findings may be currently relevant.

In the MDG era, global targets focussed on ensuring contacts with health systems, in particular on ensuring all women had at least one ANC visit. More recently, in the SDG era many have recognized the importance of ensuring good quality care, demanding a refocussing on the adequacy of content of care.

## Supporting information

S1 Fig A conceptual framework adapted from the “Right to Health” framework, Kroeger’s Model, and Good’s models for health care seeking behavior for antenatal care (ANC). (TIF)

S2 Fig Development of a thematic framework; the indexes used for women, health providers’ and health officials’ interviews and field notes. (TIF)

S1 Table A table comparing PAPFAM with MICS. (DOCX)

S2 Table The association between some of the women’s socio-demographic characteristics and receiving adequate content of ANC in Aleppo and latakia in 2006 (Phase 3). (DOCX)

S3 Table Public and private health sector differences in adequacy of ANC content in Aleppo and Latakia among women who sought ANC at a health facility (Phase 3). (DOCX)
