# Supplementary figures and images for: A mixed methods analysis of factors affecting antenatal care content: A Syrian case study
Source: PLoS One. 2019 Mar 25;14(3):e0214375. doi: 10.1371/journal.pone.0214375 (PMC6433263; doi:10.1371/journal.pone.0214375)

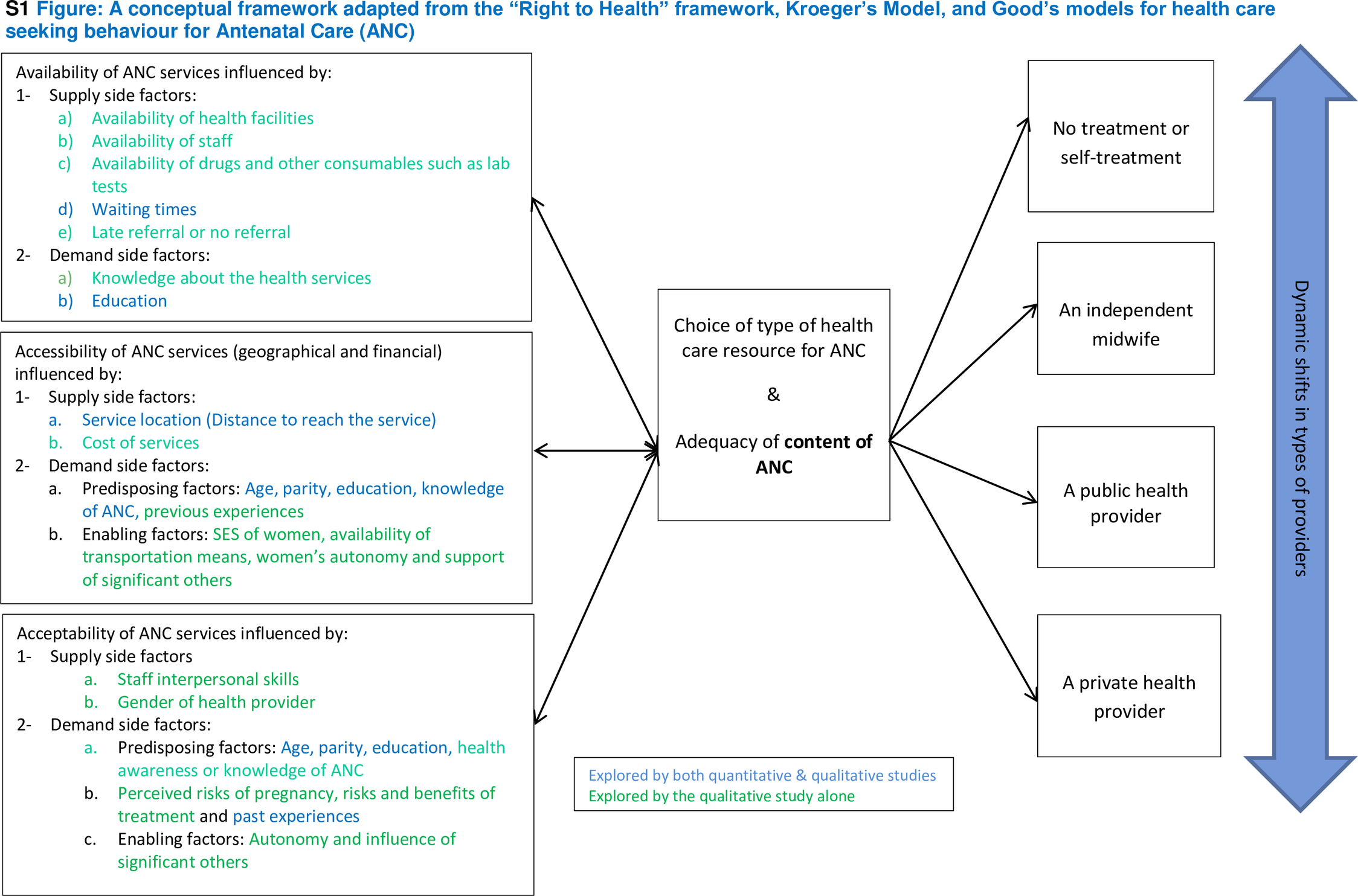

Supplement: S1 Fig — (TIF) [file pone.0214375.s001.tif]

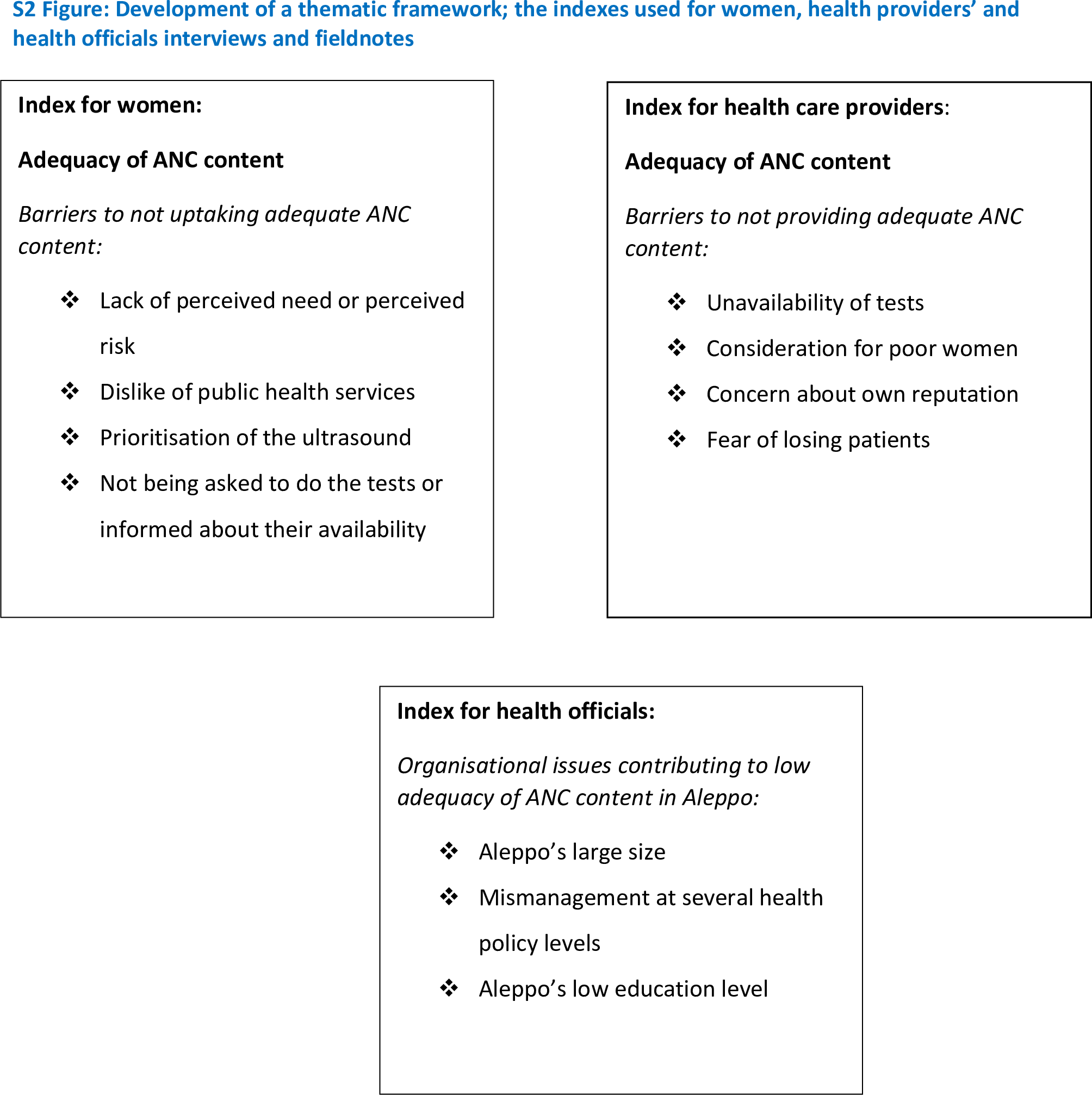

Supplement: S2 Fig — (TIF) [file pone.0214375.s002.tif]
