# Supplementary material for: A mixed methods analysis of factors affecting antenatal care content: A Syrian case study
Source: PLoS One. 2019 Mar 25;14(3):e0214375. doi: 10.1371/journal.pone.0214375 (PMC6433263; doi:10.1371/journal.pone.0214375)
Supplement: S1 Table — (DOCX) [file pone.0214375.s003.docx]

**S1. Table. A table comparing PAPFAM with MICS**

|  | PAPFAM (1995-2001) | MICS (2004-2006) |
| --- | --- | --- |
| Description | The Pan Arab Project for Family Health (PAPFAM 2001) is a national survey that was conducted in Syria in 2001 | The Multiple Indicator Cluster Survey of the Syrian Arab Republic (MICS 2006) is a national survey that was conducted in Syria in 2006. |
| Sampling design | A multi-stage stratified sample in which the country was divided into three strata: governorate centres, the remaining cities and rural areas. A total of 10,000 households were sampled. | A two-stage, stratified cluster sampling approach was used. In the first stage, clusters (primary selection units), which are census areas, were drawn in proportion to the size of the population in each governorate. The total number of selected clusters was 1000 clusters. In the second stage, households were drawn randomly from within the selected clusters. |
| Administration | The survey was administered by staff at the Central Bureau of Statistics in Damascus, Syria. | The survey was administered by staff at the Central Bureau of Statistics in Damascus, Syria. |
| Interviewed women | The reproductive health questionnaires were applied to all currently or previously married women aged between 15 and 49 years. | The reproductive health questionnaires were applied to all currently or previously married women aged between 15 and 49 years. |
| The questionnaires | The reproductive health questionnaires included questions about the mothers’ pregnancy and delivery in the **five years preceding the survey**. | The reproductive health questionnaires included questions about the mothers’ pregnancy and delivery in the **two years preceding the survey**. |
| **The availability of outcome measures** |  |  |
| **Sought ANC yes/no** | Yes | Yes |
| **First ANC visit in first trimester** | Yes | No |
| **Number of ANC visits** | Yes | No |
| **Weight measurement** | Yes | Yes |
| **Blood pressure measurement** | Yes | Yes |
| **Blood sample** | Yes | Yes |
| **Urine sample** | Yes | Yes |
| **The availability of independent variables** |  |  |
| **Age** | Yes | Yes |
| **Education** | Yes | Yes |
| **Wealth quintiles** | Computed by authors using principal component analysis | Yes |
| **Residence (rural/urban)** | Yes | Yes |
| **Governorate** | Yes | Yes |
| **Parity (children ever born)** | Yes | Yes |
| **Woman’s work status** | Yes | No |
| **Woman who reads the news paper** | Yes | No |
| **Woman who had a child who died** | No | Yes |
| **Health complications** | Yes | No |
| **Long term illness** | Yes | No |
| **Previous abortion** | Yes | No |
| **Desired pregnancy** | No | Yes |
| **Age at marriage** | Yes | Yes |
| **Husband’s age** | Yes | No |
| **Husband’s education** | Yes | No |
| **Husband’s work status** | Yes | No |
| **Problem with the unavailability of a female health provider** | Yes | No |
| **Type of health facility attended (public/private)** | Yes | Yes |
