# Supplementary material for: A mixed methods analysis of factors affecting antenatal care content: A Syrian case study
Source: PLoS One. 2019 Mar 25;14(3):e0214375. doi: 10.1371/journal.pone.0214375 (PMC6433263; doi:10.1371/journal.pone.0214375)
Supplement: S2 Table — (DOCX) [file pone.0214375.s004.docx]

**S2 Table. The association between some of the women’s socio-demographic characterisitcs and receiving adequate content of ANC in Aleppo and latakia in 2006 (phase 3)**

|  | **Aleppo (N=577)** | | | | | | | | | | | | |  |
| --- | --- | --- | --- | --- | --- | --- | --- | --- | --- | --- | --- | --- | --- | --- |
| **Women’s education** | **Secondary or higher** | | **Preparatory** | | | | **Primary** | | | | **None** | | **Total** | **Chi2 test P-value** |
| **Adequate ANC content** | 52  (64.2) | | 52  (51.5) | | | | 119 (40.5) | | | | 19 (18.8) | | 242 (41.9) | **P<0.001** |
| **Non- adequate ANC content** | 29  (35.8) | | 49  (48.5) | | | | 175 (59.5) | | | | 82  (81.2) | | 335  (58.1) |  |
| **Total** | 81 | | 101 | | | | 294 | | | | 101 | | 577 |  |
|  | **Latakia (N=144)** | | | | | | | | | | | | |  |
| **Women’s education** | **Secondary or higher** | | **Preparatory** | | | | **Primary** | | | | **None** | | **Total** | **Chi2 test P-value** |
| **Adequate ANC content** | 59  (100) | | 47  (92.2) | | | | 22  (84.6) | | | | 6  (75.0) | | 134 (93.1) | **-** |
| **Non- adequate ANC content** | 0 | | 4  (7.8) | | | | 4  (15.4) | | | | 2  (25.0) | | 10  (6.9) |  |
| **Total** | 59 | | 51 | | | | 26 | | | | 8 | | 144 |  |
|  | **Aleppo (N=577)** | | | | | | | | | | | | |  |
| **Area of residence** | **Urban** | | | | | **Rural** | | | | | | | **Total** | **Chi2 test P-value** |
| **Adequate ANC content** | 195  (47.0) | | | | | 47  (29.0) | | | | | | | 242 (41.9) | **P<0.001** |
| **Non- adequate ANC content** | 220  (53.0) | | | | | 115  (71.0) | | | | | | | 335 (58.1) |  |
| **Total** | 415 | | | | | 162 | | | | | | | 577 |  |
|  | **Latakia (N=144)** | | | | | | | | | | | | |  |
| **Area of residence** | **Urban** | | | | | **Rural** | | | | | | | **Total** | **Chi2 test P-value** |
| **Adequate ANC content** | 62  (86.1) | | | | | 72  (100) | | | | | | | 134 (93.1) | **-** |
| **Non- adequate ANC content** | 10  (13.9) | | | | | 0 | | | | | | | 10  (6.9) |  |
| **Total** | 72 | | | | | 72 | | | | | | | 144 |  |
|  | **Aleppo (N=577)** | | | | | | | | | | | | |  |
| **Socio-economic status** | **Richest** | **Fourth** | | | **Middle** | | **Second** | | | **Poorest** | | | **Total** | **Chi2 test P-value** |
| **Adequate ANC content** | 80  (55.6) | 63 (48.8) | | | 46 (38.3) | | 22 (34.9) | | | 31  (25.6) | | | 242 (41.9) | **P<0.001** |
| **Non- adequate ANC content** | 64  (44.4) | 66  (51.2) | | | 74 (61.7) | | 41 (65.1) | | | 90  (74.4) | | | 335 (58.1) |  |
| **Total** | 144 | 129 | | | 120 | | 63 | | | 121 | | | 577 |  |
|  | **Latakia (N=144)** | | | | | | | | | | | | |  |
| **Socio-economic status** | **Richest** | **Fourth** | | | **Middle** | | **Second** | | | **Poorest** | | | **Total** | **Chi2 test P-value** |
| **Adequate ANC content** | 32  (100) | 34 (100) | | | 33 (80.5) | | 28 (93.3) | | | 7  (100) | | | 134 (93.1) | **-** |
| **Non- adequate ANC content** | 0 | 0 | | | 8  (19.5) | | 2  (6.7) | | | 0 | | | 10  (6.9) |  |
| **Total** | 32 | 34 | | | 41 | | 30 | | | 7 | | | 144 |  |
|  | **Aleppo (N=577)** | | | | | | | | | | | | |  |
| **Parity level** | **1** | | | **2-3** | | | | | **>3** | | | **Total** | | **Chi2 test P-value** |
| **Adequate ANC content** | 71  (52.2) | | | 98  (43.4) | | | | | 73  (34.0) | | | 242  (41.9) | | **P=0.008** |
| **Non- adequate ANC content** | 65  (47.8) | | | 128  (56.6) | | | | | 142  (66.0) | | | 335  (58.1) | |  |
| **Total** | 136 | | | 226 | | | | | 215 | | | 577 | |  |
|  | **Latakia (N=144)** | | | | | | | | | | | | |  |
| **Parity level** | **1** | | | **2-3** | | | | **>3** | | | | **Total** | | **Chi2 test P-value** |
| **Adequate ANC content** | 32  (94.1) | | | 71  (92.2) | | | | 31  (93.9) | | | | 134  (93.1) | | **-** |
| **Non- adequate ANC content** | 2  (5.9) | | | 6  (7.8) | | | | 2  (6.1) | | | | 10  (6.9) | |  |
| **Total** | 34 | | | 77 | | | | 33 | | | | 144 | |  |
