# Supplementary material for: A mixed methods analysis of factors affecting antenatal care content: A Syrian case study
Source: PLoS One. 2019 Mar 25;14(3):e0214375. doi: 10.1371/journal.pone.0214375 (PMC6433263; doi:10.1371/journal.pone.0214375)
Supplement: S3 Table — (DOCX) [file pone.0214375.s005.docx]

**S3 Table. Public and private health sector differences in adequacy of ANC content in Aleppo and Latakia among women who sought ANC at a health facility (phase 3)**

|  | **Adequate ANC content among women who attended ANC (MICS 2006) N (%)** | | | | | | | |
| --- | --- | --- | --- | --- | --- | --- | --- | --- |
|  | **Aleppo (N=540)** | **P-value** | |  | | **Latakia (N=132)** | | **P-value** |
| **Private sector (N=420)** | 174 (41.4) | P=0.323 | | **Private sector (N=120)** | | 115 (95.8) | | **-** |
| **Public sector**  **(N=120)** | 58 (48.3) |  |  | **Public sector**  **(N=12)** | | 8 (66.7) | |  |
| **Total (N=540)** | **232 (43.0)*** |  | | **Total (N=132)** | | **123 (93.2)**** | |  |
|  | **Adequate clinical tests among women who attended ANC (MICS 2006)** | | | | | | | |
|  | **Aleppo (N=540)** | **P-value** | |  | **Latakia (N=132)** | | **P-value** | |
| **Private sector (N=420)** | 306 (72.9) | P=0.085 | | **Private sector (N=120)** | 116 (96.7) | | - | |
| **Public sector (N=120)** | 100 (83.3) |  |  | **Public sector**  **(N=12)** | 11 (91.7) | |  |  |
| **Total (N=540)** | 406 (75.2) |  | | **Total (N=132)** | 127 (96.2) | |  | |
|  | **Adequate laboratory tests among women who attended ANC (MICS 2006)** | | | | | | | |
|  | **Aleppo (N=540)** | **P-value** | |  | **Latakia (N=132)** | | **P-value** | |
| **Private sector (N=420)** | 183 (43.6) | P=0.494 | | **Private sector (N=120)** | 115 (95.8) | | **-** | |
| **Public sector (N=120)** | 58 (48.3) |  |  | **Public sector**  **(n=12)** | 8 (66.7) | |  |  |
| **Total (N=540)** | 241 (44.6) |  | | **Total (N=132)** | 123 (93.2) | |  | |
|  | **Adequate ANC content among women who started ANC early and made at least four visits (PAPFAM 2001)** | | | | | | | |
|  | **Aleppo (N=191)** | **P-value** | |  | **Latakia (N=125)** | | **P-value** | |
| **Private sector (N=164)** | 52 (31.7) | - | | **Private sector (N=112)** | 80 (71.4) | | - | |
| **Public sector (N=27)** | 4 (14.8) |  |  | **Public sector (N=11)** | 8 (72.7) | |  |  |
| **Total (N=191)** | 56 (29.3) |  | | **Total (N=123)** | 88 (71.5) | |  | |
|  | **Adequate clinical tests among women who started ANC early and made at least four visits (PAPFAM 2001)** | | | | | | | |
|  | **Aleppo (N=191)** | **P-value** | |  | **Latakia (N=125)** | | **P-value** | |
| **Private sector (N=164)** | 120 (73.2) | **P=0.005** | | **Private sector (N=112)** | 103 (92.0) | | - | |
| **Public sector (N=27)** | 11 (40.7) |  |  | **Public sector (N=11)** | 10 (90.9) | |  |  |
| **Total (n=191)** | 131 (68.6) |  | | **Total (N=123)** | 113 (91.9) | |  | |
|  | **Adequate laboratory tests among women who started ANC early and made at least four visits (PAPFAM 2001)** | | | | | | | |
|  | **Aleppo (N=191)** | **P-value** | |  | **Latakia (N=125)** | | **P-value** | |
| **Private sector (N=164)** | 59 (36.0) | P=0.166 | | **Private sector (N=112)** | 83 (74.1) | | - | |
| **Public sector (N=27)** | 6 (22.2) |  |  | **Public sector (N=11)** | 9 (81.8) | |  |  |
| **Total (N=191)** | 65 (34.0) |  | | **Total (N=123)** | 92 (74.8) | |  | |
|  | **Multivariable analysis (MICS 2006): The odds of receiving non- adequate clinical tests in Aleppo** | | | | | | | |
|  | **Adjusted †OR (95% CI)** | | **Covariate adjustment** | | | | **P-value** | |
| **Private sector** | 1.00 | | 1,2,3,4,5 | | | | **P=0.020** | |
| **Public sector** | 0.44 (0.21-0.92) | |  |  |  |  |  |  |
|  | **Multivariable analysis (MICS 2006): The odds of receiving non- adequate laboratory tests in Aleppo** | | | | | | | |
|  | **Adjusted †Odds**  **Ratio (95% CI)** | | **Covariate adjustment** | | | | **P-value** | |
| **Private sector** | 1.00 | | 1,2,3,4,5 | | | | **P=0.029** | |
| **Public sector** | 0.52 (0.29-0.93­­) | |  |  |  |  |  |  |

†Odds Ratio adjusted for (1) woman’s age, (2) education, (3) area of residence, (4) socio-economic status and (5) parity.

* 10 women who received adequate care in Aleppo attended care at both sectors so they were dropped from the analysis

** 11 women who received adequate care in Latakia attended care at both sectors so they were dropped from the analysis
